# Retinal vasculitis with Chronic Recurrent Multifocal Osteomyelitis: a case report and review of the literature

**DOI:** 10.1186/s41927-019-0076-5

**Published:** 2019-08-01

**Authors:** Victoria K. Shanmugam, Marc Phillpotts, Timothy Brady, Monica Dalal, Shawn Haji-Momenian, Esma Akin, Kavita Nataranjan, Sean McNish, Donald S. Karcher

**Affiliations:** 1 Division of Rheumatology, The George Washington University School of Medicine and Health Sciences, 2300 M Street, NW, Washington, DC 20037 USA; 2 Department of Ophthalmology, The George Washington University School of Medicine and Health Sciences, 2150 Pennsylvania Avenue NW, Washington, DC 20037 USA; 3 Department of Radiology, The George Washington University School of Medicine and Health Sciences, 2150 Pennsylvania Avenue NW, Washington, DC 20037 USA; 4 Department of Hematology, The George Washington University School of Medicine and Health Sciences, 2150 Pennsylvania Avenue NW, Washington, DC 20037 USA; 5 Department of Pathology, The George Washington University School of Medicine and Health Sciences, 2150 Pennsylvania Avenue NW, Washington, DC 20037 USA

**Keywords:** Case report, Retinal Vasculitis, Sternoclavicular, Chronic recurrent multifocal osteomyelitis, CRMO, SAPHO

## Abstract

**Background:**

Concurrent presentation of retinal vasculitis with mixed sclerotic and lytic bone lesions is rare.

**Case presentation:**

We present the case of a 37-year old woman with a several year history of episodic sternoclavicular pain who presented for rheumatologic evaluation due to a recent diagnosis of retinal vasculitis. We review the differential diagnosis of retinal vasculitis, along with the differential diagnosis of mixed sclerotic and lytic bone lesions. Ultimately, bone marrow biopsy confirmed diagnosis of chronic recurrent multifocal osteomyelitis (CRMO). Concurrent presentation of CRMO with retinal vasculitis is extremely rare but important to recognize. The patient demonstrated clinical response to prednisone and tumor necrosis factor-alpha inhibition (TNF-i).

**Conclusion:**

This case reports and unusual presentation of CRMO spectrum disease involving the sternum and sternoclavicular joint with concurrent retinal vasculitis.

## Background

Concurrent presentation of retinal vasculitis with mixed sclerotic and lytic bone lesions is rare. In this manuscript we present a case of retinal vasculitis with mixed sclerotic and lytic bone lesions and review the differential diagnosis.

## Case presentation

### Chief symptom

A 37-year-old Caucasian woman with a several year history of episodic sternoclavicular pain presented for rheumatologic evaluation following a recent diagnosis of retinal vasculitis.

### History of the present illness

**Fig. 1:**
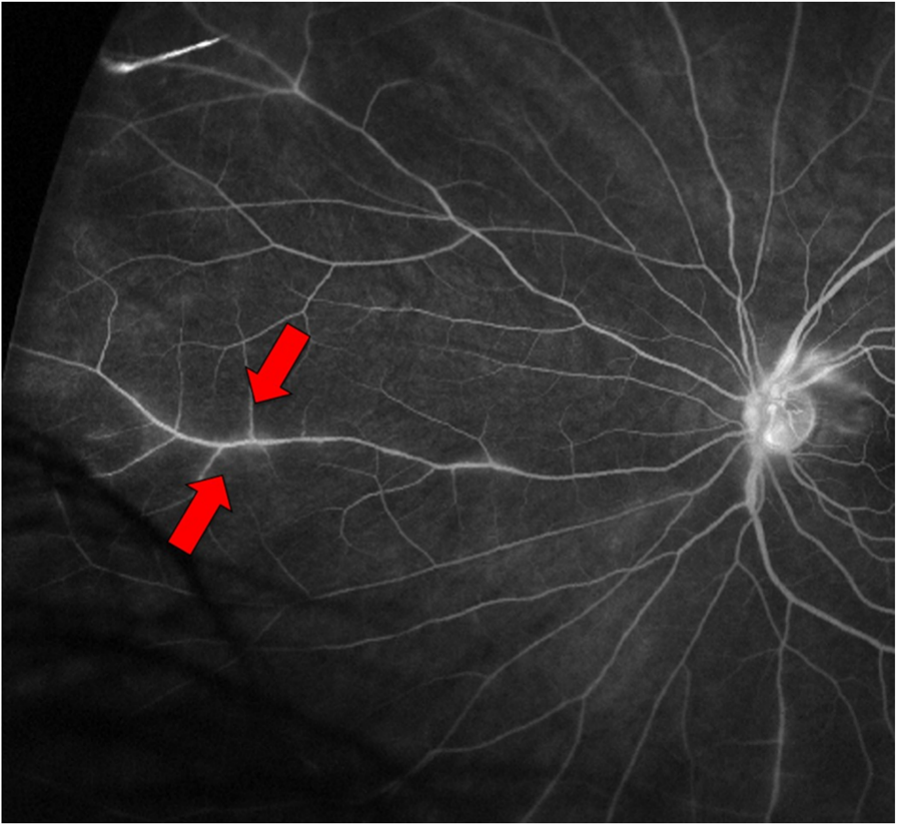
Fluorescein angiogram of the left eye demonstrating regions of retinal vascular staining and leakage near the optic nerve and in the peripheral retina (arrows)

The patient had presented to ophthalmology clinic approximately 5weeks prior to the rheumatologic evaluation with complaints of floaters in the left eye. She denied eye pain, erythema, diplopia or color desaturation. Dilated examination of the right eye demonstrated an old hyperpigmented lesion in the nasal periphery. There were no hemorrhages, other active lesions, or vascular sheathing. Dilated exam of the left eye revealed a cotton wool spot just temporal to the optic nerve. There was no associated hemorrhage or other lesions. Fluorescein angiography of the right eye demonstrated small vessel drop out in the nasal periphery, blockage from hyperpigmented lesion, and several areas of vascular staining and hyperfluorescence most notable along the superior temporal arcade. The left eye demonstrated vascular staining and hyperfluorescence most notable in the superior and nasal periphery. These findings were consistent with retinal vasculitis of both eyes (Fig. 1).

### Past medical history

Six years prior to the current presentation, a rheumatologist had evaluated the patient for sternal pain responsive to non-steroidal anti-inflammatory drugs (NSAIDs) attributed to costochondritis. At that time, CT revealed sternal sclerosis and hyperostosis with apparent periosteal reaction and erosion. MRI showed enhancement within the sternum with periostitis and cortical thickening. A sternal biopsy was performed. This showed reactive bone changes (thickened bony trabeculae) with fibrosis and occasional plasma cells, but no evidence of malignancy. The patient was told she had “costochondritis” and continued regular NSAID use.

At the time of the current presentation, the sternoclavicular pain was still present and now occurring 1–2 times per week, but still responsive to ibuprofen 600 mg orally. Her only other medication was a daily multivitamin.

The patient reported fatigue and unintentional 10-pound weight loss over the previous 6 months. She denied fevers, chills, night sweats, inflammatory low back pain, morning stiffness, inflammation in the ears or nose cartilage, epistaxis, sinusitis, cough, dyspnea, hemoptysis, wheezing or stridor. She had no history of acne, psoriasis or other pustular skin lesions. She denied history of tendonitis or enthesitis. At the time of presentation, she was actively pursuing treatment for primary infertility but had no other past medical history or relevant family or social history.

### Physical examination

On examination vital signs including body mass index were normal. The patient was alert and oriented to time, place and person. Her eye movements were conjugate. She had no scalp lesions. There was no alopecia. She had no malar or discoid rashes. Her oral aperture was normal, and she had normal salivary pooling. There were no oral ulcers. She had no cervical or axillary lymphadenopathy.

On examination of the chest wall, she had tenderness and enlargement of the right medial clavicular head, and the sternocostal junction. She had normal chest wall expansion; there was no dullness to percussion. Auscultation of the chest revealed normal air entry bilaterally with no crackles or wheeze. Abdominal examination revealed no masses; she had no hepatosplenomegaly and no lymphadenopathy. Bowel sounds were active.

Musculoskeletal examination revealed redness, erythema and warmth of the right sternoclavicular joint. There was no synovitis of the metacarpophalangeal (MCP), proximal interphalangeal (PIP), or distal interphalangeal (DIP) joints, wrists, elbows, shoulders, hips, knees, ankles or metatarsophalangeal (MTP) joints. She had full range of motion of all joints. There were no skin rashes, and she had no edema.

### Laboratory evaluation

Laboratory testing performed at the initial rheumatologic evaluation is listed in Table 1. At the time of presentation, the complete blood count was unremarkable. Inflammatory markers were elevated with C-reactive protein of 78.3 mg/L and erythrocyte sedimentation rate of 95 mm/hr. Serologic evaluation including anti-nuclear antibodies (ANA), anti-double stranded DNA antibodies (dsDNA), anti-extractable nuclear antibodies (ENA) and anti-neutrophil cytoplasmic antibodies (ANCA) were all negative. Alkaline phosphatase was within normal limits at 63 IU/L (normal range 39–117 IU/L) and vitamin D level was 45.7 ng/mL (normal range 30–100 ng/mL).

**Table 1:** Laboratory results at time of current presentation

Laboratory test	Normal range	Result at presentation
White cell count, ×1,000/μL	3.4–10.8	10.4
Hemoglobin, g/dl	11.1–15.9	10.1
Hematocrit, %	34.0–46.6	31.8
Platelet count, ×1,000/μL	150–379	467
Sodium, mmoles/liter	134–144	138
Potassium, mmoles/liter	3.5–5.2	4.3
Blood urea nitrogen, mg/dl	8–27	14
Creatinine, mg/dl	0.57–1.00	0.73
Glucose, mg/dl	65–99	94
Calcium, mg/dl	8.7–10.2	9.1
Homocysteine, μmol/l	0–15	8.8
QuantiFeron Gold	Negative	Negative
c-ANCA	Negative	Negative
p-ANCA	Negative	Negative
Atypical p-ANCA	Negative	Negative
Anti-myeloperoxidase (MPO), units/mL	0–3.5	0
Anti-proteinase-3 (PR-3), units/mL	< 9.0	< 9.0
Angiotensin converting enzyme (ACE), units/L	14–82	35
Erythrocyte sedimentation rate (ESR), mm/hr	0–32	**95**
C-Reactive protein, mg/L	0–4.9	**78.3**
Uric acid, mg/dl	2.5–7.1	4.3
Anti-streptolysin O antibody titer, units	0–200	177.1
Beta-2 glycoprotein I IgG antibody, units	< 9	< 9
Beta-2 glycoprotein I IgM antibody, units	< 9	< 9
Beta-2 glycoprotein I IgA antibody, units	< 9	< 9
Anti-cardiolipin IgG antibody, units	< 9	< 9
Anti-cardiolipin IgM antibody, units	< 9	< 9
Anti-cardiolipin IgA antibody, units	< 9	< 9
Lupus anticoagulant	Negative	Negative
Fluorescent Treponemal Antibody Absorpition	Negative	Negative
Bartonella henselae antibodies	Negative	Negative
Batronella quintana antibodies	Negative	Negative
Toxoplasma gondi antibodies	Negative	Negative
Rheumatoid factor, units/mL	0–13.5	7.6
Anti-nuclear antibody	Negative	Negative
Anti-double stranded DNA antibody	Negative	Negative
Anti-RNP antibody	Negative	Negative
Anti-Scl70 antibody	Negative	Negative
Anti-SSA antibody	Negative	Negative
Anti-SSB antibody	Negative	Negative
Anti-chromatin antibody	Negative	Negative
Anti-Jo-1 antibody	Negative	Negative
Anti-centromere antibody	Negative	Negative
Ferritin, ng/mL	15–150	51
Lactate dehydrogenase (LDH), IU/L	119–226	164
HIV	Negative	Negative
Hepatitis C	Negative	Negative
Hepatitis B	Negative	Negative
Alkaline phosphatase, IU/L	39–117	63
Total protein, g/dL	6.0–8.5	7.7
Albumin, g/dL	3.5–5.5	4.2
Aspartate aminotransferase (AST), IU/L	0–40	22
Alanine aminotransferase (ALT), IU/L	0–32	26
Vitamin D, ng/mL	30–100	45.7
HLA-B*27	Negative	Negative
Quantiferon Gold	Negative	Negative

Abnormal values are indicated in bold

Anti-cardiolipin IgM antibody was borderline at 29 mPL U/mL (normal 0–12 mPL U/mL), anti-cardiolipin IgG and IgA were negative. Lupus anticoagulant and beta-2 glycoprotein I testing were also negative. Angiotensin converting enzyme level was within normal limits.

### Imaging

Computed tomography (CT) of the thorax-performed six-years prior to the current presentation demonstrated marked abnormality in the manubrium sternum and mid-sternal body, with diffuse sclerosis and hyperostosis along with regions of periosteal reaction and erosion. Magnetic resonance imaging (MRI) of the sternum performed at that time demonstrated diffuse edema throughout the marrow of the sternum and manubrium, along with heterogeneous enhancement of the lower half of the sternum. There was adjacent soft tissue edema and enhancement, as well as cortical thickening and indistinct left-sided inframammary lymphadenopathy. Sternoclavicular joint involvement was not noted at that time, and erosions and synovitis were absent.

**Fig. 2:**
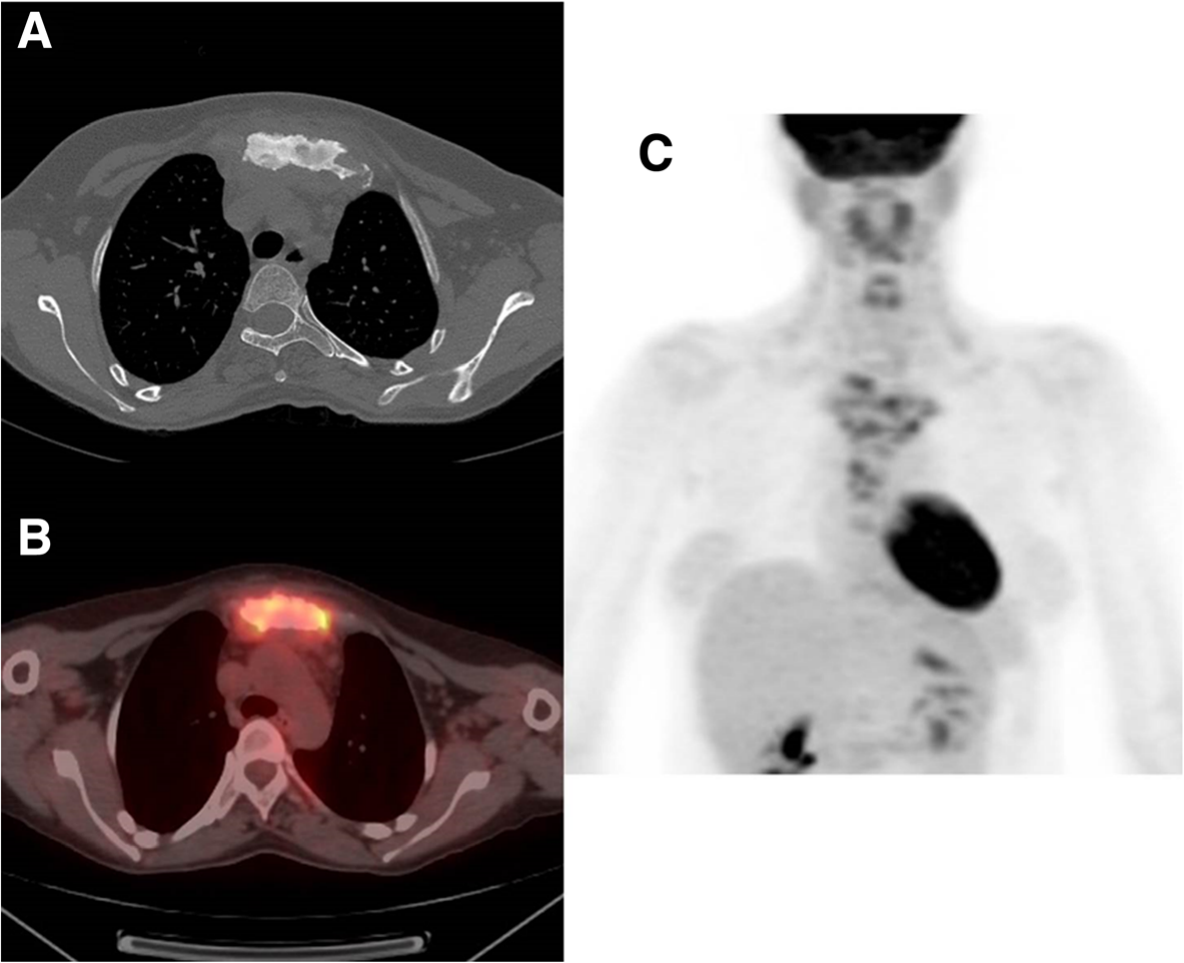
**a** Computed tomography (CT) scan of the thorax demonstrating sclerosis of sternum and manubrium with erosions of sternoclavicular joint. **b** Positron Emission Tomography - Computed Tomography (PET-CT) fusion images demonstrating multiple areas of abnormal hypermetabolism with diffuse uptake throughout the entire manubrium sternum with expansion of the manubrium and intense activity at the manubrial sternal junction. **c** Positron Emission Tomography (PET) demonstrating multiple abnormal regions of hypermetabolism corresponding with sclerotic and partially lytic lesions in the sternum and bilateral clavicular heads. Normal physiologic tracer uptake is present in the brain, heart, salivary and parotid glands, adenoids and palatine tonsils and vocal cords. There is also physiologic urinary clearance of the tracer into the renal collecting system

Initial imaging at the time of the current presentation included plain chest X-ray (Fig. 2) which demonstrated expansion and sclerosis of the sternum most visible on the lateral view as well as swelling and erosions of the sternoclavicular joint. CT of the thorax performed at the time of the current presentation demonstrated sclerosis in the manubrium sternum and sternal body. There was bony expansion and sclerosis in the medial clavicular head, extending into the metaphysis. There were small areas of lytic bone within this primary sclerotic lesion. There were multiple erosions of the sternoclavicular joint and sternocostal junction. There was inflammatory stranding in the anterior overlying subcutaneous fat. Posteriorly, inflammatory stranding extended into the anterior superior mediastinum with sub-centimeter anterior mediastinal lymph nodes present in greater number than typically seen, and a mildly enlarged left internal mammary lymph node measuring 1.1 cm (Fig. 2). Positron Emission Tomography and fusion Positon Emission Tomography-Computed Tomography (PET-CT) images demonstrated multiple areas of abnormal hypermetabolism with diffuse uptake throughout the entire manubrium sternum with expansion of the manubrium and intense activity at the manubrial sternal junction (Fig. 2).

## Differential diagnosis

This 37 year-old lady was presenting with retinal vasculitis, primary infertility and a six-year history of chronic sternoclavicular pain, elevated inflammatory markers, as well as mixed lytic and sclerotic bony expansion and erosions in the sternoclavicular joint. Presentation of retinal vasculitis with mixed sclerotic and lytic bone lesions in the sternoclavicular joint is unusual. The differential diagnosis in this case needs to take into consideration the differential diagnosis of retinal vasculitis (Table 2) and that of mixed sclerotic and lytic bone lesions (Table 3).

**Table 2:** Differential diagnosis of retinal vasculitis (diagnoses grouped by type and listed in order of frequency, those in italics are associated with retinal vasculitis so infrequently that publications are limited to case reports or very small case series [1])

Systemic diseases associated with retinal vasculitis	Infectious etiologies of retinal vasculitis	Primary ocular diseases presenting with retinal vasculitis
Behçet’s disease [2]	Cytomegalovirus [1]	Birdshot retinochoroidopathy [1]
Sarcoidosis [1, 3]	Herpes simplex virus [1]	Idiopathic Retinal Vasculitis, Aneurysms and Neuroretinitis (IRVAN) [1]
Crohn’s Disease [1]	Herpes zoster virus [1]	Eales Disease [1]
Multiple sclerosis [1]	Syphillis [1]	Vogt-Koyanagi-Harada Syndrome [4]
Systemic lupus erythematosus [1]	Tuberculosis [1]	Pars Planitis [1]
HLA-B*27 Associated [5]	Bartonella infections [6]	Retinal Arteriolitis [7]
Susac’s Syndrome [8]	Coccidioidomycosis [9]	Sympathetic ophthalmia [10]
Vasculopathy associated with antiphospholipid syndrome [11]	Human Immunodeficiency Virus (HIV) [12, 13]	
Granulomatosis with polyangiitis (GPA) [1]	Candidiasis [14]	
Eosinophilic granulomatosis with polyangiitis (EGPA) [1]	Brucellosis [15]	
Microscopic polyangiitis (MPA) [16]	Borrelia burgdorferi (Lyme disease) [17, 18]	
Polyarteritis nodosa (PAN) [1]	Leptospirosis [19]	
Giant Cell Arteritis (GCA) [20]	Rickettsia [21]	
Primary angiitis of the CNS [22]	Tropheryma Whipplei (Whipples Disease) [23]	
	Toxocariasis [24]	
	Toxoplasmosis [25]

**Table 3:** Differential diagnosis of sclerotic and lytic bone lesions

Causes of sclerotic bone lesions	Causes of lytic bone lesions	Causes of mixed sclerotic and lytic lesions
Bone islands	Fibrous dysplasia	Fibrous dysplasia
Bone infarcts	Fibrous cortical defect/ non-ossifying fibroma	Chronic recurrent multifocal osteomyelitis and SAPHO spectrum disorders
Chronic osteomyelitis	Giant Cell Tumor	Malignancy: • Breast cancer (usually sclerotic but 25% mixed) • Lung cancer (usually lytic but 25% mixed) • Testicular cancer • Cervical cancer • Prostate cancer (usually sclerotic but 15% mixed) • Ganglioneuoblastoma • Primary lymphoma of the bone
Primary bone malignancies: • Chondrosarcoma • Osteosarcoma	Metastases: • Lung cancer	Osteomyelitis
Metastases: • Prostate cancer • Breast Cancer • Other	Myeloma	Tuberculosis
Drug exposures: • Vitamin D • Fluoride	Sarcoidosis	Paget’s Disease of Bone
Fibrous Dysplasia	Aneurysmal bone cyst	Eosinophilic granuloma and Langerhans Cell Histiocytosis
Paget’s disease of the bone	Chondroblastoma	
Osteopoikilosis	Chondromyxoid fibroma	
Osteopetrosis	Hyperparathyroidism (Brown Tumor)	
Pyknodysostosis	Osteomyelitis	
Stress fracture	Intraosseous lipoma	
Hyperparathyroidism	Enchondroma	
	Eosinophilic granuloma	
	Unicameral bone cyst	

### Differential diagnosis of retinal vasculitis

Retinal vasculitis can present with blurred vision, flashes, floaters, scotoma, altered color vision, metamorphopsia (visual distortion) and pain. The term retinal vasculitis describes vascular leakage, occlusion or rupture of retinal vessels resulting in perivascular infiltrates on fundoscopic exam, which are typically confirmed on fluorescein angiography. However, these findings are not always secondary to vasculitic pathologies. Several studies have investigated etiologies of retinal vasculitic changes [1, 26, 27]. The most common rheumatic disease associated with retinal vasculitis is Behcet’s disease. However, Crohn’s Disease, sarcoidosis, systemic lupus erythematosus, HLA-B*27 associated disease, Susac’s syndrome and antiphospholipid syndrome have all been associated with retinal vasculitic injury. Additionally other systemic vasculitides including granulomatosis with polyangiitis (GPA), eosinophilic granulomatosis with polyangiitis (EGPA), microscopic polyangiitis (MPA), polyarteritis nodosa (PAN), giant cell arteritis (GCA) and primary angiitis of the central nervous system may cause retinal vasculitis relatively rarely [28]. Finally, the differential diagnosis of retinal vasculitis should also include infections and primary ocular pathologies.

### Differential diagnosis of mixed sclerotic and lytic bone lesions

The differential diagnosis of sclerotic and lytic bone lesions includes benign and malignant etiologies. However, only a small number of diseases cause chronic mixed sclerotic and lytic lesions. Etiologies of mixed sclerotic and lytic bone lesions include chronic granulomatous diseases such as sarcoidosis, eosinophilic granuloma, tuberculosis, Paget’s disease of the bone, and the SAPHO spectrum disorders including chronic recurrent multifocal osteomyelitis.

For the purposes of this review and discussion, we will focus on diseases that are known to cause both lytic/sclerotic bone lesions and retinal vasculitis.

### Autoimmune etiologies

#### Sarcoidosis

Sarcoidosis is a chronic systemic disease of unknown etiology characterized by non-caseating granulomas of affected organs. Ocular involvement with sarcoidosis is common, with 30–60% of patients eventually developing ophthalmologic disease [29, 30]. Posterior segment involvement with sarcoid affects approximately 28% of cases, most commonly with retinal periphlebitis presenting with perivascular cuffing and sheathing. More than 60% of patients with sarcoid eye disease have elevated serum angiotensin converting enzyme (ACE) level and abnormal chest imaging [29].

Musculoskeletal symptoms are common in sarcoidosis affecting 4 to 38% of sarcoid patients, and may be the first manifestation of the disease [31]. However, true sarcoid bone lesions are often associated with more severe and chronic disease. Bone lesions are typically lytic, with multiple punctate lytic lesions producing a lacy appearance, often seen in the proximal and middle phalanges of the hands [32–34]. Long bone and axial skeletal lesions may be radiographically occult, or may appear as focal either lytic or sclerotic areas. Magnetic resonance imaging can show non-specific changes including changes resembling periostitis [34]. Increased uptake may be seen on scintigraphy before lesions become clinically apparent. In this case, the presence of mixed sclerotic and lytic bone lesions as well as the absence of hilar lymphadenopathy and normal ACE level made sarcoid an unlikely diagnosis.

#### Relapsing Polychondritis

Relapsing polychondritis is an immune disorder in which there is inflammation in cartilaginous structures including the eyes, ears, nose, joints and respiratory tract. In about one-third of cases, relapsing polychondritis is associated with another connective tissue disease, systemic vasculitis, or myelodysplastic syndrome [35]. The most common ocular manifestation of relapsing polychondritis is scleritis [36]. However, retinal vasculitis has been reported. Typically, in relapsing polychondritis however the musculoskeletal manifestation is a non-erosive inflammatory arthritis, and thus the mixed sclerotic and lytic bone lesions seen in this case could not have been caused by relapsing polychondritis.

#### HLA-B27 associated arthropathies

The Human Leukocyte Antigen B27 (HLA-B27) associated arthropathies include ankylosing spondylitis and other forms of spondyloarthritis can result in bony sclerosis and ankyloses but not typically mixed sclerotic and lytic lesions. Ocular involvement in HLA-B27 associated arthropathies, can present with acute recurrent unilateral anterior uveitis. Posterior segment manifestations are also reported including vitritis, cystoid macular edema and optic disc leakage. Cases of retinal vasculitis in HLA-B27 positive patients are also reported and this can be seen associated with posterior segment involvement in 20–25% of patients [37]. In this case, HLA-B27 testing was negative, essentially excluding the HLA-B27 associated arthropathies as an etiology.

#### Behçet’s syndrome

Behçet’s Syndrome is an inflammatory disorder of unknown cause characterized by recurrent oral and genital ulcers, uveitis and skin lesions [38]. Behçet’s is one of the most common causes of retinal vasculitis in Europe [26]. This disease also commonly involves the gastrointestinal tract and can be associated with vasculitis and venous and arterial thrombotic events. Although many cases of Behçet’s are sporadic, there is a known association with the presence of HLA-B51 allele and sometimes patients have a family history of the disease. The arthritis of Behçet’s is characterized by an asymmetric, non-erosive, non-deforming inflammatory arthritis of medium and large joints including the knees, wrists and ankles [39]. However, Behçet’s does not typically cause mixed sclerotic and lytic bone lesions. Furthermore, the absence of oral and genital ulceration in this patient essentially excluded Behçet’s as a diagnosis.

### Hematologic disorders

#### Plasma cell Dyscrasias

Plasma cell dyscrasias such as multiple myeloma may present with lytic bone lesions [40]. Bone lesions are seen in 80% of patients with newly diagnosed myeloma and more than 50% of patients report bone pain at diagnosis [41, 42]. However, these lesions do not typically have sclerosis and do not show increased uptake on nuclear bone scan. The syndrome of Polyneuropathy, Organomegally, Endocrinopathy, Monoclonal Gammopathy and Skin Changes (POEMS syndrome) is associated with sclerotic bone lesions and at least one case involving lytic and sclerotic changes in the sternum has been reported [43]. However, POEMS syndrome typically presents in the 40–60 year old age group and is more common in men [44]. POEMS syndrome has not been reported to cause a retinal vasculitis. In this patient the normal serum protein electrophoresis and absence of endocrinopathy made POEMS syndrome an unlikely diagnosis.

#### Eosinophilic granuloma and Langerhans cell Histiocytosis

Langerhans Cell Histiocytosis is a rare histiocytic disorder characterized by single or multiple osteolytic bone lesions. Histiocytes may infiltrate any organ, but when patients present with mono-ostotic lytic bone lesions it is referred to as Eosinophilic Granuloma. Any bone may be involved but there is a predilection for the skull (50%), pelvis (23%), metadiaphyses of long bones, spine, ribs (8%) and mandible (7%). In adults, rib lesions are the most common site of involvement. Radiographs generally show a lytic lesion, which may be well-demarcated or have a more aggressive appearance, with periosteal reaction [45]. In the skull, lesions may give rise to 1) the “button sequestrum” in which the lytic lesion contains a central devascularized bony sequestrum, 2) the “hole within a hole” sign, in which there is uneven involvement of the inner and outer tables of the skull, or 3) the “geographic skull” characterized by large well-circumscribed lytic lesions. Alveolar destruction around the root of a tooth can also give the radiographic appearance of a “floating tooth”. Destruction and severe collapse of a vertebral body gives rise to vertebra plana. The appearance on bone scintigraphy may be variable. MRI signal characteristics can also be variable but often show contrast enhancement. Erdheim-Chester Disease (a rare histiocytic neoplasm) can cause mixed sclerotic and lytic bone lesions and intraocular involvement with choroidal infiltration and retinal detachments [46–48]. In the case presented, the mixed sclerotic and lytic lesion and its contiguous involvement across a joint did not have the typical appearance of a histiocytic lesion, but biopsy was recommended to exclude this etiology.

### Infectious diseases

#### Tuberculosis

Osteoarticular mycobacterial infections may result in inflammatory bone lesions. Worldwide, *Mycobacterium tuberculosis (TB)* is the most common cause of osteoarticular mycobacterial infection with other non-tuberculous mycobacterial infections being much rarer. Bone and joint tuberculosis accounts for 2.2% of tuberculosis cases in the United States and about 3–5% of tuberculosis cases worldwide with approximately 10% of cases involving the sacroiliac joints [49]. Tuberculosis of the sternum is rare and accounts for only 1.5% of these cases [50]. Radiographic changes in osteoarticular tuberculosis include bone demineralization, bone destruction and sequestrum formation. In TB of the sternum, computed tomography would show soft tissue swelling, irregular cortical contours and small fluid loculations with inflammatory changes in the parasternal region. Skeletal scintigraphy would show increased radiotracer uptake in the sternum and sternoclavicular joints. Magnetic resonance imaging would show signal hypointensity in cortical bone and abnormal bone marrow signal intensity (hypointense on T1-weighted images, hyperintense on T2-weighted images) [51]. These findings were absent in this case.

TB can also cause ocular manifestations with or without evidence of systemic TB [52, 53]. TB may disseminate to any part of the eye resulting in choroiditis, chorioretinitis, choroidal granuloma, optic neuritis, optic disc granuloma, subretinal abscess, orbital cellulitis, scleritis, necrotizing scleritis, posterior scleritis, scleokeratouveitis, interstitial keratitis and anterior chamber granuloma. There are also cases of ophthalmic hypersensitivity reactions in response to distant infection in which episcleritis, phlyctenulosis and occlusive retinal vasculitis develop with a pattern of occlusive retinal vasculopathy known as Eales disease. In patients with HIV, immune recovery uveitis can occur with immune reconstitution after initiation of anti-retroviral therapy. This patient had a negative QuantiFERON-TB gold screening test and no other systemic findings of tuberculosis.

#### Syphillis

Syphillis is an infection caused by the spirochete *Treponema pallidum*, and may cause inflammatory lesions in the eye and bone [54]. Posterior uveitis and panuveitis are the most common ocular manifestations of syphilis. However, it would be unusual to develop ocular syphilis without syphilitic meningitis. Bone lesions reported in syphilis infections include synovitis, osteitis and periostitis. The syphilis serology in this patient was negative and thus this was excluded as a possible diagnosis.

### Bone diseases

#### Primary lymphoma of the bone

Primary lymphoma of the bone is a rare lymphoid malignancy that accounts for 5% of primary bone tumors and 3–15% of all extra-nodal non-Hodgkin’s lymphoma [55]. The peak incidence occurs in the fifth decade with a slight male predominance (1.5:1); the disease is rare but has been reported in children and young adults [56]. Any bony site may be affected, but the femur is the most common location. On plain films, lesions are most commonly lytic, but mixed lytic and sclerotic lesions may coexist. The bone cortex may show a mixture of permeative, moth-eaten and destructive patterns with reactive periosteal changes. Typically, there will be increased uptake on ^99^technecium-MDP scanning and ^18^FDG-PET scanning. Histopathological analysis of bone lymphomas can be challenging but the most common histologic subtype accounting for 70–80% of cases is diffuse large B-cell lymphoma (DLBCL). In this case a biopsy was recommended to exclude primary lymphoma of the bone.

#### Paget’s disease of the bone

Paget’s disease of the bone is a chronic disorder of excessive bone remodeling. It is relatively common, and the prevalence increases with age. The disease is rare below the age of 55 years, but over the age of 70 it can affect up to 5% of women and 8% of men [57]. It is more common in people of European descent indicating a likely genetic component to disease pathogenesis although an infectious trigger has also been postulated based on the observation of intranuclear inclusion bodies resembling paramyxovirus in osteoclasts of pagetic bone. Many patients with Paget’s disease of the bone are identified based on elevation of serum alkaline phosphatase on a routine blood draw. However, approximately 30–40% of patients have symptoms at presentation and bone pain is the most common presenting symptom. Lesions typically progress through three phases: lytic, mixed and blastic phases, with radiographs demonstrating coarsened bony trabeculae, cortical thickening and bony expansion. Bone lesions may be monoostotic or polyostotic with a preferential involvement of the axial skeleton (pelvis in 70%, femur 55%, lumbar spine 53%, skull 42% and tibia 32% [57]). Involvement of the sternum in Paget’s disease of the bone is relatively rare but has been reported [58]. The relatively young age of this patient made Paget’s disease of the bone an unlikely diagnosis furthermore the alkaline phosphatase was within normal limits and typically this would be elevated in classic Paget’s disease of the bone.

### Autoinflammatory disorders

#### SAPHO Spectrum disorders and chronic recurrent multifocal osteomyelitis (CRMO)

The SAPHO spectrum disorders refer to a spectrum of inflammatory diseases often associated with dermatologic manifestations characterized by the pentad of synovitis, acne, pustulosis, hyperostosis and osteitis. Chronic recurrent multifocal osteomyelitis (CRMO) is now considered one of the SAPHO spectrum disorders.

CRMO is a rare autoimmune inflammatory disorder of bone [59] often presenting in childhood or adolescence (mean ± SD age 10.9 ± 2.9 years at diagnosis). It is more common in women (2:1 female: male ratio) and classically presents with recurrent flares of inflammatory bone pain and swelling. Patients typically have elevated inflammatory markers and sometimes the flares are associated with fever. Approximately 20–30% of cases are unifocal at presentation but some become multifocal over time [59]. The most common skeletal sites affected are the metaphyseal regions of tubular bones, but in unifocal disease the mandible and clavicle are also common sites. Lesions can have lytic and sclerotic appearance and approximately one quarter of patients with multifocal disease have symmetric involvement [60, 61].

## Diagnostic testing

In order to complete investigative work up, both bone marrow biopsy and biopsy of the sternal lesion was performed.

### Bone marrow biopsy

The bone marrow biopsy revealed high normocellular bone marrow, with mild megakaryocyte hyperplasia, absent iron stores, and mild plasmacytosis. Flow cytometric analysis of the aspirate specimen revealed no immunophenotypic evidence of abnormal hematopoiesis or increase in myeloblasts. The marrow findings suggested that the thrombocytosis was reactive in nature. Immunohistochemical and in-situ hybridization (ISH) stains were performed to evaluate for a clonal population of plasma cells. CD138 stain (a marker of plasma cells and multiple myeloma cells) showed plasma cells representing 10–15% of marrow elements. The plasma cells were noted to occur mostly individually, with small aggregates adjacent to small blood vessels. The plasma cells were negative for CD56 (a marker of NK cells). Kappa:lambda ratio among the plasma cells was approximately 2:1. These findings were consistent with a polyclonal population of plasma cells.

#### Sternum and sternoclavicular joint biopsies

**Fig. 3:**
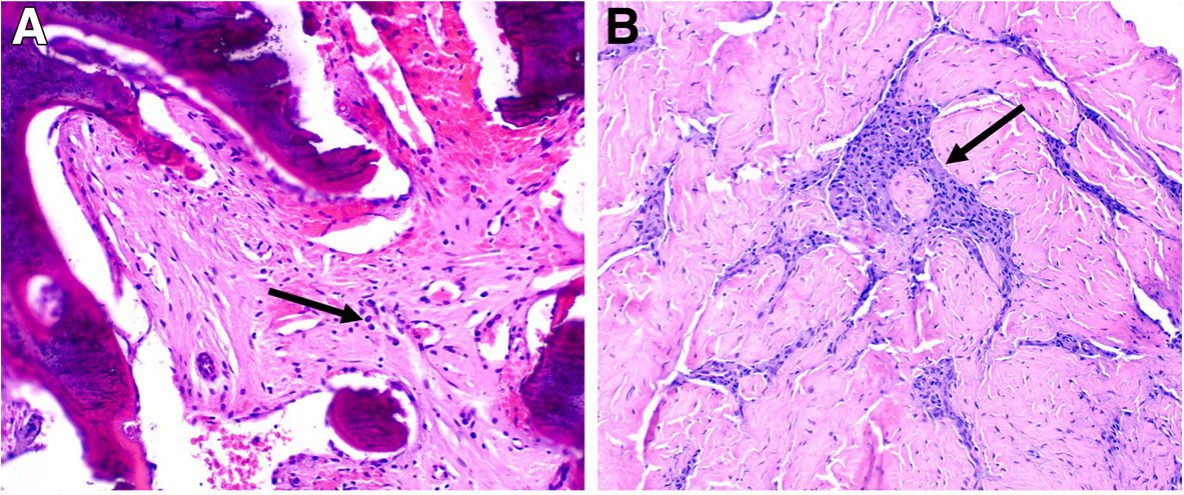
**a** Sternum biopsy showing thickened bony trabeculae (incompletely decalcified during tissue processing) and fibrosis in the medullary cavity with small collections of plasma cells (arrow) and scattered lymphocytes (H&E stain, X100). **b** Sternoclavicular joint biopsy showing dense fibrous connective tissue, presumed to be the joint capsule, with focal collections of lymphocytes and plasma cells (arrow). (H&E stain, X100)

Given the concerns for malignancy, biopsies of the sternum and the right sternoclavicular joint were obtained. Pathologic examination of the sternum biopsy (Fig. 3a) revealed thickened bony trabeculae and fibrosis in the medullary cavity, with small collections of plasma cells and small lymphocytes. The sternoclavicular joint biopsy (Fig. 3b) showed dense fibrous connective tissue, presumed to be joint capsule, with focal collections of lymphocytes and a few plasma cells. No evidence of neoplasm was identified in either specimen.

## Diagnosis

Given the presence of hyperostosis and chronic inflammatory changes on the biopsy with absence of primary lymphoma or other malignancy a diagnosis of chronic recurrent multifocal osteomyelitis with concurrent retinal vasculitis was made.

## Discussion and conclusion

This case demonstrates an unusual initial presentation of CRMO spectrum disorder involving the sternum and sternoclavicular joint with concurrent retinal vasculitis. This association has not previously been reported. While clavicular involvement in CRMO may be seen in up to 25% of cases, most lesions start medially and spare the sternoclavicular joint [62]. The aggressive appearance and soft tissue inflammation in this case, as well as involvement of the sternoclavicular joint raised concerns for a malignant process. However, bone and joint biopsies ruled out malignant and other diagnoses, and demonstrated reactive bone changes with chronic inflammatory findings consistent with CRMO [63].

Current management of CRMO includes NSAIDS for pain control, immunosuppression with traditional DMARDs including sulfasalazine and methotrexate [59, 64], tumor necrosis factor-alpha (TNF-α) inhibitors [65] and bisphosphonates [66]. A small study has shown the combination of infliximab with methotrexate and zolendronic acid is effective at reducing disease activity [67].

Prednisone was initiated with rapid improvement in her pain and inflammatory markers. Plans were in progress to commence a TNF-α inhibitor as a steroid sparing agent when the patient conceived naturally. During the pregnancy, she was treated with prednisone 20 mg daily. At 34 weeks gestation she had spontaneous rupture of membranes and delivered a healthy baby girl via cesarean section (due to breech presentation). There were no post-partum complications. After completion of lactation, she continued to have elevated inflammatory makers and significant pain and swelling at the sternoclavicular joint. TNF-α inhibitor therapy was initiated with adalimumab (Humira®) and prednisone was slowly tapered. Her bone lesion has clinically responded to this therapy with reduction in pain and inflammation and marked improvement in her inflammatory markers.

The final diagnosis in this case was that of chronic recurrent multifocal osteomyelitis with retinal vasculitis. Inflammatory eye syndromes including episcleritis have previously been reported in association with CRMO spectrum disorders [68]. However, in this case we describe a novel report of retinal vasculitis with confirmed evidence of CRMO on bone biopsy adding to the literature of the association between CRMO spectrum disorders and inflammatory eye disease.

## Data Availability

Data sharing is not applicable to this article since no datasets were generated or analyzed during this study.

## Abbreviations

ACE: Angiotensin converting enzyme
ANA: Antinuclear antibody
CRMO: Chronic recurrent multifocal osteomyelitis
DIP: Distal interphalangeal joint
DLBCL: Diffuse large B-Cell lymphoma
DMARD: Disease modifying antirheumatic therapy
dsDNA: double stranded DNA
EGPA: Eosinophilic granulomatosis with polyangiitis
FDG-PET: Fluordeoxyglucose-Positron emission tomography
GCA: Giant cell arteritis
GPA: Granulomatosis with polyangiitis
HLA-B27: Human leucocyte antigen B27
MCP: Metacarpophalangeal joint
MPA: Microscopic polyangiitis
MRI: Magnetic resonance imaging
NSAID: Non-steroidal anti-inflammatory drug
PAN: Polyarteritis nodosa
PIP: Proximal interphalangeal joint
SAPHO: Synovitis, acne, pustulosis, hyperostosis and osteitis
TB: Tuberculosis
TNFi: Tumor necrosis factor-inhibitor
